# Mitochondrial biomarkers predict tumor progression and poor overall survival in gastric cancers: Companion diagnostics for personalized medicine

**DOI:** 10.18632/oncotarget.19962

**Published:** 2017-08-05

**Authors:** Federica Sotgia, Michael P. Lisanti

**Affiliations:** ^1^ Translational Medicine, School of Environment & Life Sciences, Biomedical Research Centre, University of Salford, Greater Manchester, United Kingdom

**Keywords:** gastric cancer, mitochondrial biomarkers, overall survival, tumor progression

## Abstract

Here, we employed a bioinformatics approach to identify novel molecular determinants to predict tumor progression and overall survival in gastric cancer patients. In particular, we directly assessed whether nuclear-derived mRNA species encoding proteins involved in mitochondrial protein translation and OXPHOS are able to successfully predict clinical outcome in gastric cancer. As such, using *in silico* validation, we have now established the prognostic value of these mitochondrial biomarkers, in a defined population of gastric cancer patients. In this context, we interrogated 5 year follow-up data collected from a group of N = 359 gastric cancer patients. Importantly, in this group of cancer patients, Ki67 and PCNA (conventional markers of cell proliferation) were associated with tumor progression, as might be expected. Using this simplified informatics approach, we identified ∼75 new individual mitochondrial gene probes that effectively predicted tumor progression, with hazard-ratios (HR) of up to 2.22 (*p* < 2.1e-10). These mitochondrial mRNA transcripts included heat shock proteins/chaperones, membrane proteins, anti-oxidants, enzymes involved in genome maintenance, as well as mitochondrial ribosomal proteins (MRPs) and numerous members of the OXPHOS complexes. In addition, we combined 8 mitochondrial protein transcripts (NDUFS5, VDAC3, ATP5O, IMMT, MRPL28, COX5B, MRPL52, PRKDC), to generate a compact gastric mitochondrial gene signature, associated with a HR of 2.77 (*p* = 1.4e-14). As a result of this analysis and validation, we strongly suggest that proteins involved in mitochondrial protein translation and OXPHOS should be considered as targets for new drug discovery, for the treatment of gastric cancers. The mitochondrial markers we identified here could also be used as companion diagnostics, to predict clinical outcomes, as well as the patient response to therapy. This should allow a more successful and personalized approach to gastric cancer diagnosis and therapy.

## INTRODUCTION

Gastric cancer, also known as stomach cancer, starts as an infectious disease that drives the development of chronic inflammation and ulcerated lesions, located within the stomach wall lining. In most cases of gastric cancer, these lesions are morphologically defined as epithelial carcinomas [1–3]. During tumor progression, the gastric cancer cells migrate and metastasize to the lymph nodes, the abdomen, bones, lungs and the liver. The most common etiology of gastric cancer is chronic infection with *Helicobacter pylori*, a bacterium, which represents ∼50-70% of the cases [4–6]. Classic symptoms consist of “heartburn”, abdominal pain, nausea and vomiting, as well as anemia and occult blood in the feces. The diagnosis of gastric cancer is confirmed by an endoscopic biopsy, revealing an invasive carcinoma. The disease is most common in Asian countries, especially in South Korea and Japan, among others [1–6].

Unfortunately, the 5-year survival rates for gastric cancer patients vary widely, from as low as 15% to as high as 60%. As such, more effective biomarkers are urgently needed for the early stratification of gastric cancer patients, into low-risk and high-risk groups at diagnosis. Here, we directly tested the hypothesis that markers of mitochondrial biogenesis and respiratory function may have significant prognostic value in the early identification of high-risk gastric cancer patients, with increased tumor progression and poor overall survival.

More specifically, we employed a bioinformatics approach to determine the prognostic utility of nuclear-encoded mitochondrial mRNA species in predicting clinical outcome. Our results indicate that ∼75 individual mitochondrial gene probes can be effectively used, to predict progression and survival in gastric cancer patients. As a result, we propose the idea that mitochondria are novel therapeutic targets for drug discovery, aimed at optimizing the effectiveness of gastric cancer therapy [7–9].

## RESULTS

### Prognostic value of proliferative and inflammatory markers in gastric cancer patients

**Figure 1:**
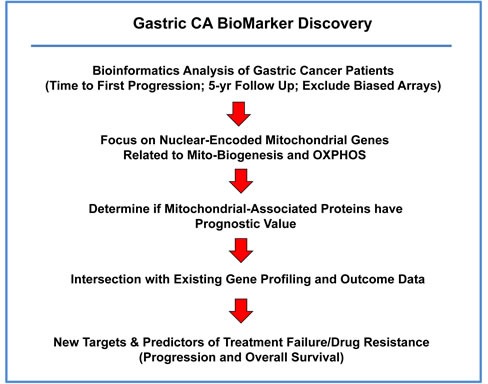
A bio-informatics approach to gastric cancer marker discovery We chose to focus on gastric cancer patients, with 5-years of follow-up data (*N* = 359). In this context, we evaluated the prognostic value of mitochondrial markers for predicting time to first progression and overall survival.

**Figure 2:**
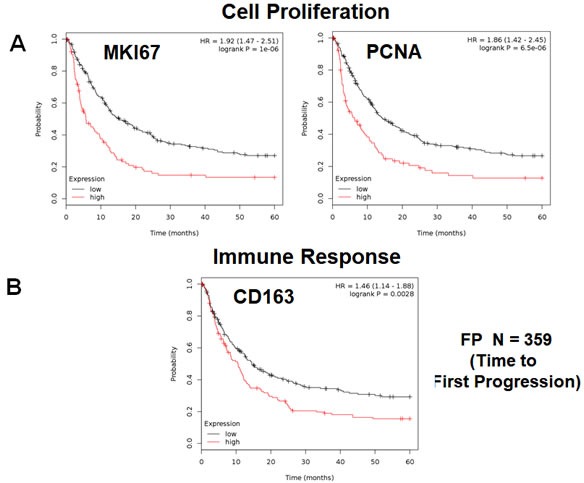
Markers of proliferation and inflammation predict poor clinical outcome in gastric cancer patients We assessed the predictive value of Ki67 and PCNA in *N* = 359 gastric cancer patients. **A.** Note that high transcript levels of Ki67 and PCNA are associated with significantly reduced time to first progression. Note that the official gene name for the Ki67 protein is MKI67. **B.** Note that that high transcript levels of CD163 are associated with significantly reduced time to first progression.

Here, we used publically available transcriptional profiling data from the tumors of gastric cancer patients, with 5 years of follow-up data, to identify new potential biomarkers (Figure 1). As proliferative biomarkers are often used as clinical endpoints in patient trials, we first assessed the prognostic value of Ki67 and PCNA, in this patient population. Tables 1, 2 and Figure 2 both show the prognostic value of these markers. The hazard-ratios for Ki67 and PCNA were 1.79 and 1.86, respectively, for time to first progression (FP).

**Table 1:** Prognostic value of Ki67 in gastric cancer

Gene Probe ID	Symbol	Hazard-Ratio	Log-Rank Test
212020_s_at	MKI67	1.92	1e-06
212023_s_at	MKI67	1.92	2.7e-07
212022_s_at	MKI67	1.83	1.9e-06
212021_s_at	MKI67	1.60	0.002
**Combined**		**1.79**	**6.2e-06**

**Table 2:** Prognostic value of PCNA and CD163 in gastric cancer

Gene Probe ID	Symbol	Hazard-Ratio	Log-Rank Test
217400_at	PCNA	1.86	6.5e-06
216233_at	CD163	1.46	0.003

We also assessed the prognostic value of a macrophage-specific marker of inflammation, namely CD163. Table 2 and Figure 2 show that CD163 effectively predicted time to first progression, with a hazard-ratio of 1.46. Thus, conventional markers of proliferation and inflammation predict overall survival in gastric cancer patients.

### Prognostic value of individual mitochondrial markers

To evaluate the hypothesis that elevated mitochondrial mass, biogenesis and function somehow contributes towards poor clinical outcome in gastric cancer patients, we also determined the prognostic value of individual mitochondrial markers.

**Figure 3:**
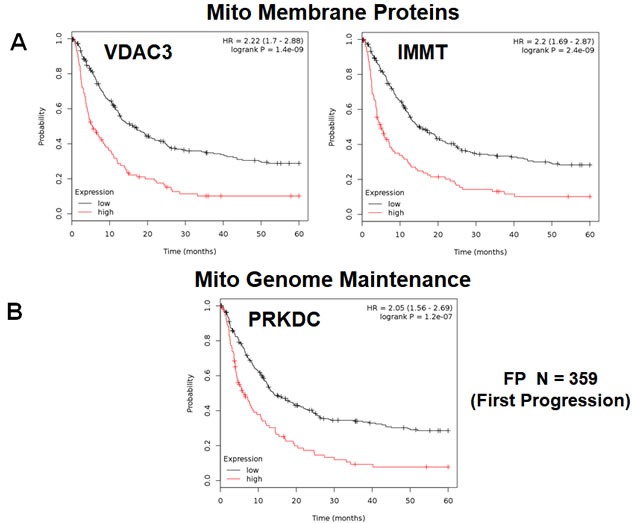
Mitochondrial membrane proteins and PRKDC are associated with poor clinical outcome in gastric cancer patients **A.** Note that that high transcript levels of VDAC3 and IMMT are associated with significantly reduced time to first progression. **B.** Note that that high transcript levels of PRKDC are associated with significantly reduced time to first progression.

First, we examined the behavior of mitochondrial membrane proteins and HSPs/chaperones. Table 3 and Figure 3 both show that VDAC3 and IMMT had the best prognostic value, with a hazard-ratio of up to 2.22 (p<1.4e-09). HSPD1 and members of the TIMM and TOMM gene families also had prognostic value; SOD2 also had significant value. Positive results were also obtained with DNA-PK (PRKDC), a kinase that maintains the integrity and copy number of the mitochondrial genome (mt-DNA).

**Table 3:** Prognostic value of mitochondrial HSPs and other mitochondrial proteins

Gene Probe ID	Symbol	Hazard-Ratio	Log-Rank Test
**Heat Shock Proteins and Chaperones (4 probes)**			
200807_s_at	HSPD1	1.83	1.9e-06
200806_s_at	HSPD1	1.56	0.003
200691_s_at	HSPA9	1.61	0.0002
205565_s_at	FXN	1.38	0.01
**Membrane Proteins (9 probes)**			
208844_at	VDAC3	2.22	1.4e-09
211662_s_at	VDAC2	1.51	0.002
200955_at	IMMT	2.20	2.4e-09
218118_s_at	TIMM23	1.91	4.2e-07
218408_at	TIMM10	1.88	1.4e-06
218357_s_at	TIMM8B	1.49	0.002
201821_s_at	TIMM17A	1.33	0.025
201870_at	TOMM34	1.95	5.1e-07
202264_s_at	TOMM40	1.44	0.009
**Mitochondrial Anti-Oxidants (2 probes)**			
215223_s_at	SOD2	1.72	2.1e-05
215078_at	SOD2	1.70	2.9e-05
**Mitochondrial Genome Maintenance (3 probes)**			
208694_at	PRKDC	2.05	1.2e-07
210543_s_at	PRKDC	1.78	6.9e-06
215757_at	PRKDC	1.47	0.003

**Figure 4:**
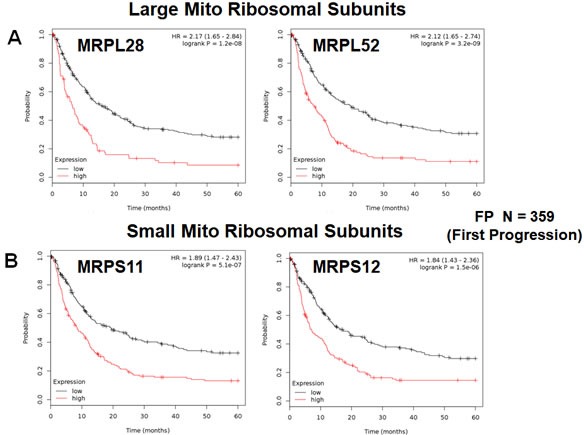
Mitochondrial ribosomal proteins (MRPs) are associated with poor clinical outcome in gastric cancer patients **A.** Note that high transcript levels of MRPL28 and MRPL52 predict significantly reduced time to first progression. **B.** Similarly, high transcript levels of MRPS11 and MRPS12 predict significantly reduced time to first progression.

Next, we determined the prognostic value of mitochondrial ribosomal proteins (MRPs), which are essential for mitochondrial biogenesis (Table 4). Twelve components of the large subunit (MRPLs) showed significant prognostic value, with hazard-ratios between 2.17 and 1.29. Notably, MRPL28 had the best prognostic value. Eight different components of the small subunit (MRPSs) showed significant prognostic value, with hazard-ratios between 1.89 and 1.36. As a result, twenty different MRPs all predicted reduced time to first progression. Representative examples of Kaplan-Meier curves are shown in Figure 4, panels A & B.

**Table 4:** Prognostic value of mitochondrial ribosomal proteins

Gene Probe ID	Symbol	Hazard-Ratio	Log-Rank Test
**Large Ribosomal Subunit (12 probes)**			
204599_s_at	MRPL28	2.17	1.2e-08
221997_s_at	MRPL52	2.12	3.2e-09
222216_s_at	MRPL17	1.68	0.0001
220527_at	MRPL20	1.67	0.0002
217907_at	MRPL18	1.62	0.0004
218887_at	MRPL2	1.60	0.0002
203931_s_at	MRPL12	1.56	0.001
208787_at	MRPL3	1.53	0.0007
217919_s_at	MRPL42	1.52	0.002
218049_s_at	MRPL13	1.47	0.008
218281_at	MRPL48	1.40	0.009
213897_s_at	MRPL23	1.29	0.049
**Small Ribosomal Subunit (8 probes)**			
215919_s_at	MRPS11	1.89	5.1e-07
213840_s_at	MRPS12	1.84	1.5e-06
210008_s_at	MRPS12	1.47	0.004
204330_s_at	MRPS12	1.37	0.015
204331_s_at	MRPS12	1.37	0.037
203800_s_at	MRPS14	1.53	0.002
219220_x_at	MRPS22	1.44	0.005
219819_s_at	MRPS28	1.42	0.01
218112_at	MRPS34	1.36	0.02

**Figure 5:**
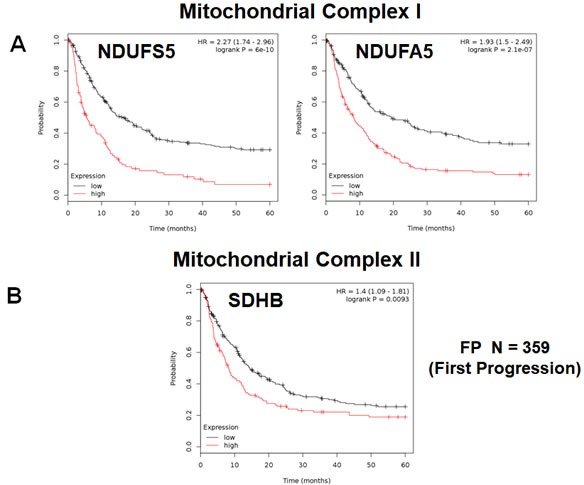
Mitochondrial complex I and II proteins are associated with poor clinical outcome in gastric cancer patients **A.** Note that high levels of NDUFS5 and NDUFA5 predict significantly reduced time to first progression. **B.** Similarly, high levels of SDHB predict significantly reduced time to first progression.

**Figure 6:**
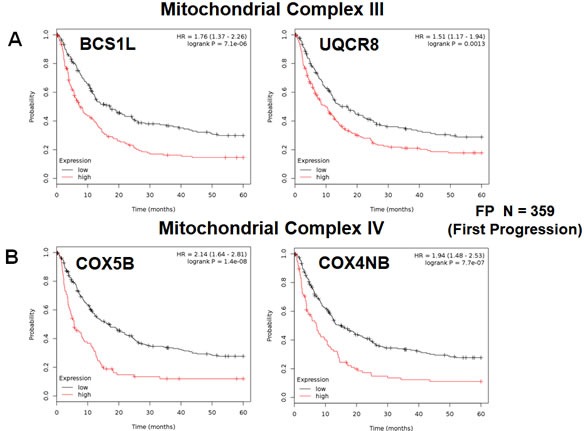
Mitochondrial complex III and IV proteins are associated with poor clinical outcome in gastric cancer patients **A.** Note that high levels of BCS1L and UQCR8 predict significantly reduced time to first progression. **B.** Similarly, high levels of COX5B and COX4NB predict reduced time to first progression.

**Figure 7:**
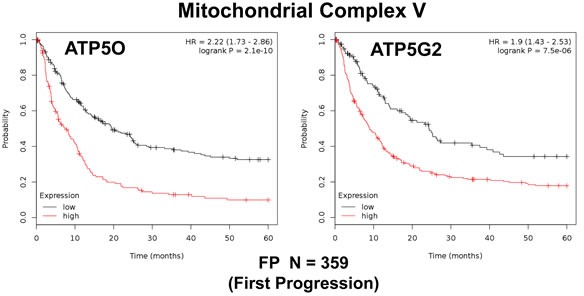
Mitochondrial complex V proteins are associated with poor clinical outcome in gastric cancer patients Note that high levels of ATP5O and ATP5G2 predict significantly reduced time to first progression.

In addition, we evaluated the prognostic value of members of the OXPHOS complexes I-V. These results are summarized in Table 5. Interestingly, 37 different gene probes for the OXPHOS complexes showed hazard-ratios between 2.27 and 1.30. NDUFS5 (complex I) had the best prognostic value (HR=2.27; p=6e-10). COX5B (complex IV) and ATP5O (complex V) also showed significant prognostic value (HR=2.14; p=1.4e-08 *versus* HR=2.22; p=2.1e-10). Kaplan-Meier analysis for components of complex I and II are shown in Figure 5A, 5B, while results with complex III and IV are shown in Figure 6A, 6B. Results with complex V are highlighted in Figure 7.

**Table 5:** Prognostic value of mitochondrial OXPHOS complexes

Gene Probe ID	Symbol	Hazard-Ratio	Log-Rank Test
**Complex I (11 probes)**			
201757_at	NDUFS5	2.27	6e-10
215850_s_at	NDUFA5	1.93	2.1e-07
208969_at	NDUFA9	1.92	1.5e-06
203606_at	NDUFS6	1.74	7.9e-05
214241_at	NDUFB8	1.67	5.7e-05
203371_s_at	NDUFB3	1.51	0.002
218226_s_at	NDUFB4	1.49	0.003
202001_s_at	NDUFA6	1.37	0.02
218160_at	NDUFA8	1.31	0.04
202785_at	NDUFA7	1.31	0.04
218563_at	NDUFA3	1.30	0.04
**Complex II (1 probe)**			
214166_at	SDHB	1.40	0.009
**Complex III (2 probes)**			
207618_s_at	BCS1L	1.76	7.1e-06
202233_s_at	UQCR8	1.51	0.001
**Complex IV (10 probes)**			
213736_at	COX5B	2.14	1.4e-08
218057_x_at	COX4NB	1.94	7.7e-07
201754_at	COX6C	1.74	7.1e-05
201441_at	COX6B1	1.67	0.0001
200925_at	COX6A1	1.64	8.8e-05
203880_at	COX17	1.60	0.0003
217451_at	COX5A	1.49	0.006
202110_at	COX7B	1.42	0.01
217249_x_at	COX7A2	1.33	0.035
216003_at	COX10	1.33	0.046
**Complex V (13 probes)**			
221677_s_at	ATP5O	2.22	2.1e-10
207552_at	ATP5G2	1.90	7.5e-06
207335_x_at	ATP5I	1.84	8.4e-06
217801_at	ATP5E	1.64	0.0002
208972_s_at	ATP5G1	1.51	0.002
210149_s_at	ATP5H	1.49	0.003
202961_s_at	ATP5J2	1.47	0.004
210453_x_at	ATP5L	1.45	0.006
207573_x_at	ATP5L	1.44	0.01
208746_x_at	ATP5L	1.40	0.009
213366_x_at	ATP5C	1.33	0.03
206993_at	ATP5S	1.29	0.04
213366_x_at	ATP5C1	1.33	0.03

### Prognostic value of a short gastric (Ga) mito-signature

To increase the prognostic significance of these unique mitochondrial markers, we then combined the most promising markers to create a new gastric mitochondrial gene signature.

**Figure 8:**
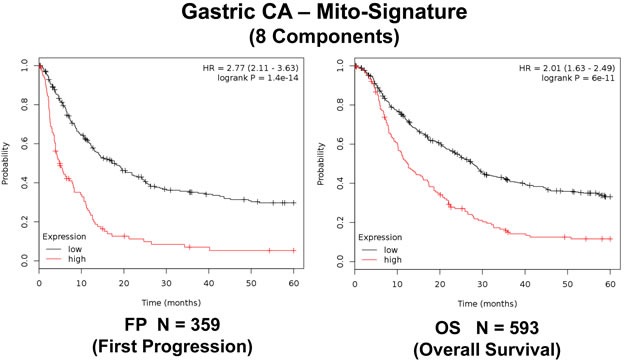
A short mitochondrial signature predicts tumor progression and overall survival in gastric cancer patients We combined 8 mitochondrial proteins (NDUFS5, VDAC3, ATP5O, IMMT, MRPL28, COX5B, MRPL52, PRKDC), to generate a compact gastric mitochondrial gene signature.

More specifically, Ga-Mito-Signature-1 contains 8 genes (NDUFS5, VDAC3, ATP5O, IMMT, MRPL28, COX5B, MRPL52, PRKDC). Importantly, Ga-Mito-Signature-1 yielded a significantly improved hazard-ratio for time to first progression (FP) (HR=2.77; p=1.4e-14), as well as for overall survival (OS) (HR=2.01; p=6e-11). These results are summarized in Table 6 and highlighted in Figure 8.

**Table 6:** Compact gastric CA mito-signature for predicting clinical outcome

Gene Probe ID	Symbol	Hazard-Ratio	Log-Rank Test
201757_at	NDUFS5	2.27	6e-10
208844_at	VDAC3	2.22	1.4e-09
221677_s_at	ATP5O	2.22	2.1e-10
200955_at	IMMT	2.20	2.4e-09
204599_s_at	MRPL28	2.17	1.2e-08
213736_at	COX5B	2.14	1.4e-08
221997_s_at	MRPL52	2.12	3.2e-09
208694_at	PRKDC	2.05	1.2e-07
**Combined**		**2.77**	**1.4e-14**

As such, the Ga-Mito-Signature-1 represents a significant improvement over individual mitochondrial biomarkers, as well as Ki67 and PCNA.

## DISCUSSION

### New “druggable” targets for inhibiting mitochondrial biogenesis and blocking oxidative metabolism in cancer cells

Here, we suggest that the new mitochondrial markers that we developed here may also be useful for selecting new “druggable” targets for innovative drug development, to prevent treatment failure and improve overall survival. As a consequence of our K-M analyses, we believe that the mitochondrial ribosome would be an attractive new target for developing novel inhibitors of mitochondrial protein translation in cancer cells; similarly, mitochondrial chaperones, the OXPHOS complexes and the mitochondrial ATP-synthase may also be suitable drug targets. Particular components of these multi-subunit protein complexes show significant prognostic value, suggesting that modulation of their intrinsic activity may provide therapeutic benefits. Targeting of these large protein complexes would be predicted to suppress tumor recurrence and prevent disease progression in these gastric cancer patients.

**Figure 9:**
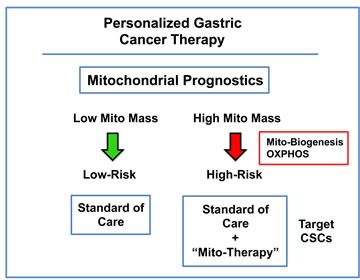
Gastric cancer: Mitochondrial-based diagnostics for personalized therapy In this proposed scheme, mitochondrial markers could be used to separate gastric cancer patients into high-risk and low-risk groups. Then, patients with high levels of mitochondrial markers in their primary tumor (“bad prognosis”) would be treated with mitochondrial-based therapies, to prevent progression and increase survival.

Similarly, these mitochondrial markers could also be employed as companion diagnostics for novel therapies targeting either mitochondrial protein translation or cellular respiration, to select the high-risk sub-population of gastric cancer patients, resulting in the proper treatment stratification (Figure 9). In direct support of this assertion, we showed here that ∼75 different mitochondrial probes could be used to successfully identify the sub-population of high-risk gastric cancer patients. As such, these results indicate that mitochondrial markers could be used to monitor and/or predict the response to therapy, identifying patients at high-risk for treatment failure at diagnosis, several years in advance, even prior to the initiation of therapy.

### Cancer stem cells (CSCs) have increased mitochondrial power: diagnostic and therapeutic implications

Recently, we identified increased mitochondrial function as a new feature of cancer stem cells (CSCs), based on unbiased proteomics analysis [7–9,11]. This increase is mitochondrial function in CSCs may be secondary to increased mitochondrial biogenesis and/or elevated mitochondrial protein translation, resulting in the steady-state amplification of mitochondrial mass [7–9,11]. These findings have important implications for designing new approaches for identifying and eradicating CSCs.

Importantly, our previous studies directly show that MitoTracker (a fluorescent mitochondrial vital dye) can be used, together with flow cytometry, to purify CSCs from heterogeneous populations of cancer cells [12,13]. Importantly, the “Mito-high” cells showed the greatest functional capacity for i) anchorage-independent growth and ii) tumor-initiating activity *in vivo* [12–14]. Quantitatively similar results were also obtained with other mitochondrial probes (for detecting ROS and H2O2) and/or NAD(P)H auto-fluorescence, all surrogate markers of increased mitochondrial respiratory activity [14].

In support of these functional studies, we have also shown that FDA-approved antibiotics can be used to effectively target mitochondria in CSCs [15–21]. Because mitochondria evolved from aerobic bacteria, over a period >1.5 billion years, certain types of antibiotics functionally inhibit mitochondrial biogenesis, as a manageable side-effect. In this context, tetracycline antibiotics, such as Doxycycline, appeared to be the most promising. For example, Doxcycline effectively eradicates CSCs in 12 different cell lines, representing 8 different cancer types, as well as in primary tissue samples derived from patients with advanced metastatic disease (collected from pleural effusions or ascites fluid) [15,16].

Interestingly, these functional observations could mechanistically explain the high prognostic value of mitochondrial markers in predicting progression and survival in gastric cancer patients. Patients with high levels of mitochondrial markers may have increased levels of CSCs (Figure 9). As a result, these mitochondrial biomarkers could also be used as companion diagnostics, to identify which gastric cancer patients would benefit most from anti-cancer therapy with Doxycycline (or perhaps with other inhibitors of mitochondrial biogenesis and/or drugs metabolically targeting respiratory function, such as Metformin).

## METHOD OF ANALYSIS

Kaplan-Meier (K-M) Analyses. To perform K-M analysis on nuclear mitochondrial gene transcripts, we used an open-access, online survival, data analysis tool to interrogate publically available microarray data from up to 1,065 gastric cancer patients [10]. This allowed us to determine their overall prognostic value. For this purpose, we primarily analyzed 5-year follow-up data from gastric cancer patients (N = 359). Biased array data were excluded from the analysis. This allowed us to identify ∼75 nuclear mitochondrial gene probes, with significant prognostic value. Hazard-ratios for time to first progression (FP) and overall survival (OS) were calculated, at the best auto-selected cut-off, and p-values were calculated using the logrank test and plotted in R. K-M curves were also generated online using the K-M-plotter (as high-resolution TIFF files) [10], using univariate analysis:

http://kmplot.com/analysis/index.php?p=service&cancer=gastric

This allowed us to directly perform *in silico* validation of these mitochondrial biomarker candidates.

## Abbreviations

CSCs: cancer stem-like cells
HR: hazard ratio
K-M: Kaplan-Meier
MRPL: mitochondrial ribosomal proteins, large subunit
MRPS: mitochondrial ribosomal proteins, small subunit
N: number of patients in a given data set
OS: overall survival
OXPHOS: oxidative phosphorylation (mitochondrial respiration)
FP: time to first progression
